# Back to the Wastes: The Potential of Agri-Food Residues for Extracting Valuable Plant Cell Wall Polysaccharides

**DOI:** 10.3390/ijms26104942

**Published:** 2025-05-21

**Authors:** Susana Saez-Aguayo, Dayan Sanhueza, Paloma Fuenzalida, María Paz Covarrubias, Michael Handford, Raúl Herrera, María Alejandra Moya-León

**Affiliations:** 1 Centro de Biotecnología Vegetal, Laboratorio Mucilab, Facultad de Ciencias de la Vida, Universidad Andrés Bello, Santiago 8370146, Chile; dayansanhueza@gmail.com; 2 ANID-Anillo de Investigación en Ciencia y Tecnología-Chilean Fruits Cell Wall Components as Biotechnological Resources (CHICOBIO) ACT210025, Talca 3460000, Chile; paloma.fuenzalida@ug.uchile.cl (P.F.); mariapaz.covarrubias@gmail.com (M.P.C.); mhandfor@uchile.cl (M.H.); raherre@utalca.cl (R.H.); 3 ANID-Millennium Science Initiative Program-Millennium Nucleus for the Development of Super Adaptable Plants (MN-SAP), Santiago 8320211, Chile; 4 Centro de Biología Molecular Vegetal, Departamento de Biología, Facultad de Ciencias, Universidad de Chile, Las Palmeras 3425, Ñuñoa, Santiago 7800024, Chile; 5 Laboratorio de Fisiología Vegetal y Genética Molecular, Instituto de Ciencias Biológicas, Universidad de Talca, Talca 3460000, Chile

**Keywords:** cell wall, circular bioeconomy, fruit wastes, hemicelluloses, immunodetection, pectins, polysaccharides

## Abstract

The agro-industrial sector generates large volumes of fruit waste each year, leading to environmental concerns and sustainability challenges. In this study, we evaluate the potential of fruit residues—apple, pear, blueberry, tomato, papaya, and a mixed fruit juice blend—as alternative sources of high-value polysaccharides, including pectins, hemicelluloses, and cellulose. Additionally, white strawberry, included as a reference from fresh fruit rather than agro-industrial waste, was analyzed to expand the comparative framework. These biopolymers, naturally derived from the plant cell wall, are renewable and biodegradable, and they possess physicochemical properties suitable for applications in food, pharmaceutical, cosmetic, textile, and bioenergy industries. Using a combination of cell wall fractionation, biochemical characterization, and immunodetection of specific structural domains, we identified significant variability in polysaccharide composition and structure among the samples. Blueberry, pear, and apple residues showed high levels of rhamnogalacturonan-I (RG-I) with extensive branching, while variations in rhamnogalacturonan-II (RG-II) dimerization and the degree of methylesterification of homogalacturonan were also observed. These structural differences are key to determining the gelling properties and functional potential of pectins. In the hemicellulose fractions, xylans and xyloglucans with distinct substitution patterns were especially abundant in apple and pear waste. Our findings demonstrate that fruit processing waste holds significant promise as a sustainable source of structurally diverse polysaccharides. These results support the reintegration of agro-industrial residues into production chains and emphasize the need for environmentally friendly extraction methods to enable industrial recovery and application. Overall, this study contributes to advancing a circular bioeconomy by transforming underutilized plant waste into valuable functional materials.

## 1. Introduction

In recent years, the search for methods to reduce waste generation has become increasingly important. In particular, the agro-industrial sector produces an enormous amount of waste each year, which conflicts with the goal of creating more eco-friendly societies. Today, it is more important than ever to find new ways to give a second life to residual materials, so that what was once considered waste can be reintegrated into the production chain, in line with sustainable practices [1,2].

In this context, the waste generated by the food industry, particularly from fruit and vegetable processing, represents a source with high potential for exploitation due to the presence of various polysaccharides [3]. These polysaccharides, which include cellulose, pectins, and hemicellulose (HC), are essential components of plant cell walls, and each of them contributes with unique characteristics depending on their composition [4,5,6]. These natural polymers are abundant, renewable, and biodegradable, making them highly attractive for sustainable development in numerous industries, including food, pharmaceuticals, textiles, cosmetics, and bioenergy [7,8,9]. Their unique structural properties and functional characteristics have opened the door to innovative technologies aimed at enhancing product performance while reducing environmental impacts.

Cellulose, the most abundant biopolymer on Earth, is a key structural component of plant cell walls and is widely used in industry due to its versatility and strength. In the paper and textile industries, cellulose is processed into fibers for making paper products, fabrics, and other materials [10,11]. Beyond traditional applications, cellulose is increasingly utilized in the production of bio-based plastics, films, and coatings, where it serves as an eco-friendly alternative to petroleum-based materials. Additionally, in the pharmaceutical and food industries, cellulose derivatives like microcrystalline cellulose are employed as thickeners, stabilizers, and excipients in drug formulations and food products [12,13]. Furthermore, cellulose nanocrystals and nanofibers are gaining attention for their potential use in advanced materials such as composites, packaging, and biomedical devices due to their exceptional mechanical properties [14,15,16]. Pectins, another group of plant-derived polysaccharides, are widely used as gelling agents, stabilizers, and emulsifiers, particularly in the food and cosmetic industries. Pectins are known for their ability to form gels in the presence of sugars and acids, making them ideal for producing jams, jellies, and other food products [12,13]. In addition to their gelling properties, pectins are also used in the pharmaceutical industry for controlled drug release, in wound healing materials, and as dietary fibers in functional foods, contributing to gastrointestinal health and lowering cholesterol levels [17,18,19,20,21,22]. Their biodegradability and biocompatibility make them a promising material for biomedical applications, such as in tissue engineering and drug delivery systems [23]. Finally, HCs, a heterogeneous group of polysaccharides, are utilized in various industrial processes. In the production of biofuels, HC-rich biomass is broken down into fermentable sugars, which are then converted into ethanol or other biofuels, offering a renewable energy source [24,25,26,27]. HCs are also employed in the food industry as emulsifiers, stabilizers, and dietary fibers [28]. Furthermore, HC-derived materials are being explored for use in biodegradable packaging, hydrogels, and adhesives [28].

Therefore, the integration of plant-based polysaccharides into industrial processes highlights their potential to replace synthetic, non-renewable materials with natural and sustainable alternatives [3]. Their wide range of applications underscores the need for continued research and development to fully unlock their potential [1,2,6,8,28]. The growing interest in plant cell wall polysaccharides not only drives industrial innovation but also promotes a circular bioeconomy by transforming agro-industrial waste streams into valuable resources for polysaccharide extraction.

In this context, we analyzed the cell wall composition of six types of residues generated from the production of juice, tomato sauce, and traditional Chilean sweets, specifically focusing on blueberry, apple, pear, tomato, papaya, and mixed juice residues. Additionally, we included white strawberry—a fruit with good organoleptic quality but short postharvest shelf life [29]—as a fresh fruit reference. Although white strawberry is cultivated, it remains underexploited, making it a useful non-agri-food waste control in this study. The polysaccharide composition of the different fruit wastes was analyzed by an initial cell wall extraction, followed by cell wall fractionation, biochemical analysis, and immunodetection of various cell wall domains. The results revealed significant variability in the polysaccharide composition among the different residues. This exploration provides valuable insights for reincorporating these agro-industrial residues into a wide range of diverse production processes while minimizing environmental impacts and promoting sustainable practices.

## 2. Results

### 2.1. Analysis of Monosaccharide Composition in Alcohol-Insoluble Residues

The analysis of polysaccharide composition across samples of five fruit wastes— papaya, blueberry, pear, apple, and tomato, as well as fruit juice, and white strawberry as a fresh fruit control, reveals distinct extraction yields and sugar profiles, indicating significant variations in cell wall content and structure. First, when comparing the yield of alcohol-insoluble residues (AIR) extracted from different fruit sources, which reflects the carbohydrate contents, clear differences were detected (Supplementary Table S1). Interestingly, the yield of total polysaccharides extracted from blueberry waste was notably high, accounting for nearly half of its weight (51.6%). For the other processed-fruit wastes (apple, pear, tomato waste, and fruit juice), the yield of the total cell wall material extracted was around 20%, which is around eight times greater than the yield for fresh white strawberry fruit (Supplementary Table S1). Fresh white strawberries and papaya seed mucilage exhibit a low AIR yield (2.5% and 1.2%, respectively), which is expected given that both samples originate from fresh fruit and contain a high-water content, as they have not undergone any industrial processing.

**Figure 1:**
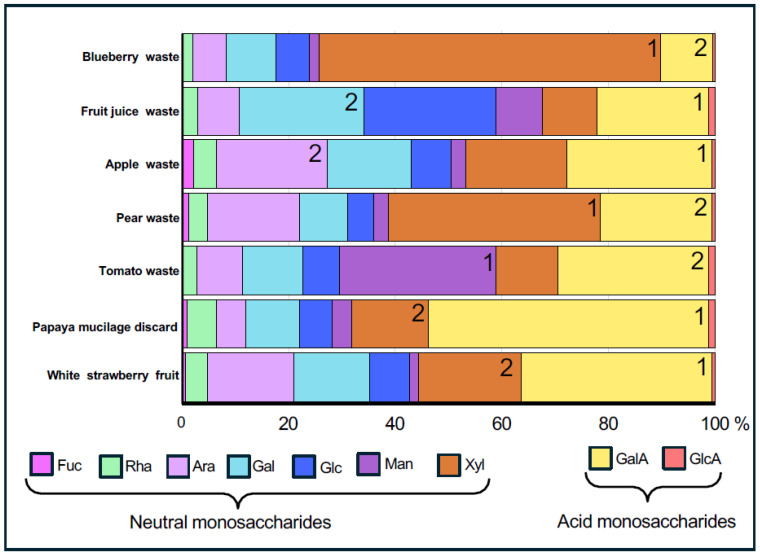
Graphical representation of the proportion of different sugars presents in alcohol-insoluble residues (AIR) after acidic hydrolysis with TFA from various fruit waste samples. AIRs were prepared from the different fruit waste samples and subjected to acidic hydrolysis using trifluoroacetic acid (TFA). The resulting monosaccharides were analyzed by high-performance anion-exchange chromatography with pulsed amperometric detection (HPAEC-PAD). The graph displays the relative abundance of each monosaccharide, expressed as a percentage of the total monosaccharide content. Monosaccharide abbreviations: Fuc, fucose; Rha, rhamnose; Ara, arabinose; Gal, galactose; Glc, glucose; Man, mannose; Xyl, xylose; GalA, galacturonic acid; GlcA, glucuronic acid. Numbers indicate the two most abundant monosaccharides in the composition.

Each AIR preparation was analyzed for its monosaccharide composition (Supplementary Table S2). Conversion coefficients from monomeric to polymeric forms were also considered, with slight adjustments made to the values; however, the relative proportions of the sugars remained unchanged (Supplementary Table S3). Each sample exhibited distinct monosaccharide proportions, indicating variability in the polysaccharide content (Figure 1). For instance, blueberry and pear wastes exhibit high Xyl levels. Tomato waste is particularly rich in Man, while fruit juice waste is remarkably rich in Glc. Papaya waste, primarily composed of seed mucilage, is characterized by an elevated GalA content. In tomato and apple waste, as well as in white strawberry, approximately 30% of their composition consists of GalA. The variation in the proportion of Ara and Gal present in the samples was also notable.

### 2.2. Chemical Separation of Pectins and Hemicelluloses from Waste Samples

**Figure 2:**
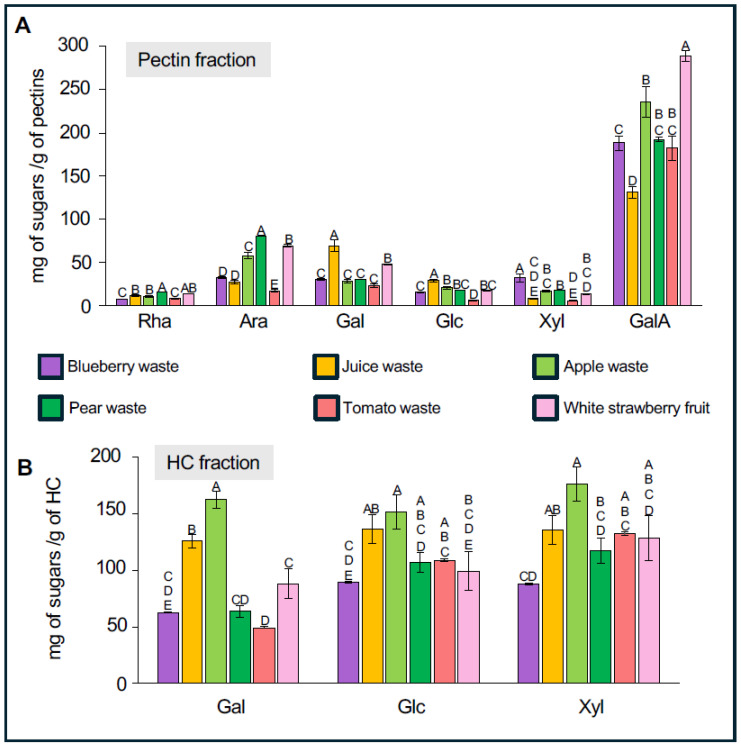
Visualization of the Predominant Monosaccharides Identified in Pectin- and Hemicellulose-Enriched Fractions. (**A**). Monosaccharide composition was determined by HPAEC-PAD after TFA hydrolysis of pectin-enriched fractions. (**B**). Monosaccharide composition was determined by HPAEC-PAD after TFA hydrolysis of HC-enriched fractions. Both graphs display the concentrations of the main monosaccharides found in pectin and hemicellulose, expressed as milligrams of monosaccharide per gram of extracted pectin or hemicellulose. The principal monosaccharides are indicated as follows: Rha, rhamnose; Ara, arabinose; Gal, galactose; Glc, glucose; GalA, galacturonic acid; Xyl, xylose (*n* = 6, from two independent extractions). Bars represent standard error (SE). Statistical analysis was performed using one-way ANOVA followed by Tukey’s post hoc test (*p* < 0.05).

The analysis of the AIRs reveals variations in monosaccharide content but remains inconclusive regarding the specific pectin or HC domains present in the residues (Figure 1). To gain deeper insights, AIR was fractionated into pectin- and hemicellulose-enriched fractions, revealing substantial variation in their yields across the analyzed fruit waste samples (Supplementary Table S1). While blueberry and tomato wastes, in addition to fruit juice, share a similar relative abundance of pectins and HC, apple and pear wastes have a higher HC proportion than pectins, and in contrast, papaya and white strawberry samples are higher in pectins content than HC (Supplementary Table S1). On the other hand, the pectin yield across these samples shows notable variability, and interestingly, papaya waste and white strawberry are rich in pectins (40% and 30% of AIR, respectively) indicating that these sources could be particularly rich in pectic polysaccharides such as HG, RG-I, and/or RG-II (Figure 2). In agreement with this, the AIR material from these two fruit sources is rich in GalA (Figure 1). The hemicellulose yield also varies across the samples, with values ranging from 13.7 to 30.7% of AIR, with pear wastes being the samples with the highest HC contribution, followed by papaya and apple wastes (Supplementary Table S1). HC polymers could include xylans, xyloglucans, or arabinoxylans, and coincident with that, AIR material from pear, papaya, and apple contains moderate-high Xyl, Glc, and Ara contents (Figure 1).

### 2.3. Analysis of the Monosaccharide Composition in Fractions Enriched in Pectin and Hemicellulose

As a way to get a better identification of the type of pectins and HC polymers present in each waste sample, the monosaccharide composition of the pectin- and HC-enriched fractions was determined (Figure 2 and Supplementary Table S2). For the pectin fraction, the most abundant monosaccharide in all samples is GalA, followed by Gal, Ara, and Rha. The highest amount of GalA was observed in white strawberry with 289.2 mg of sugars per gram of pectin (Figure 2A, Supplementary Table S2). This value is higher than those of blueberry, pear, tomato, and apple wastes, which contain around 235 mg g^−1^ of sugars. Juice waste shows the lowest GalA content at 131.2 mg g^−1^. When analyzing Rha content, white strawberry and pear waste stand out with the highest amounts: 13.7 mg g^−1^ and 16.0 mg g^−1^ of pectins, respectively. In contrast, lower Rha levels were found in juice (11.6 mg g^−1^), apple (10.9 mg g^−1^), tomato (8.1 mg g^−1^), and blueberry (7.5 mg g^−1^). High Ara levels were detected in pear (81.1 mg g^−1^), white strawberry (69.5 mg g^−1^), and apple (58.1 mg g^−1^) pectin fractions. The Gal profile differs slightly, with the highest levels found in juice waste (69.2 mg g^−1^) and white strawberry (48.1 mg g^−1^) (Figure 2A and Supplementary Table S2).

For HC-enriched fractions, the proportion of each monosaccharide (Glc, Xyl, Gal) across the different fruit wastes is relatively similar, with juice and apple waste showing the highest content of Glc, Xyl, and Gal, while blueberry waste exhibits the lowest levels of HC-related sugars (Figure 2B, Supplementary Table S2). Focusing on the main sugars of xyloglucans—Glc, Xyl, and Gal—apple waste shows concentrations of 151.3, 176.2, and 162.6 mg g^−1^ of HC, respectively. Juice waste also possesses high levels of these sugars, with 136.4 mg g^−1^ Glc, 135.8 mg g^−1^ Xyl, and 125.6 mg g^−1^ Gal. Pear, tomato, and white strawberry residues exhibit intermediate sugar levels, while blueberry waste has the lowest HC content, containing approximately three times less sugar than in pear waste. Specifically, blueberry HC fractions contain 89.4 mg g^−1^ Glc, 88.17 mg g^−1^ Xyl, and 62.5 mg g^−1^ Gal (Figure 2B and Supplementary Table S2). Interestingly HC-enriched fraction from pear and tomato wastes contains a relatively lower proportion of Gal content (49–63 mg g^−1^) than juice and apple residues (125–162 mg g^−1^).

### 2.4. Analysis of the Isolated Rhamnogalacturonan-I Domain

**Figure 3:**
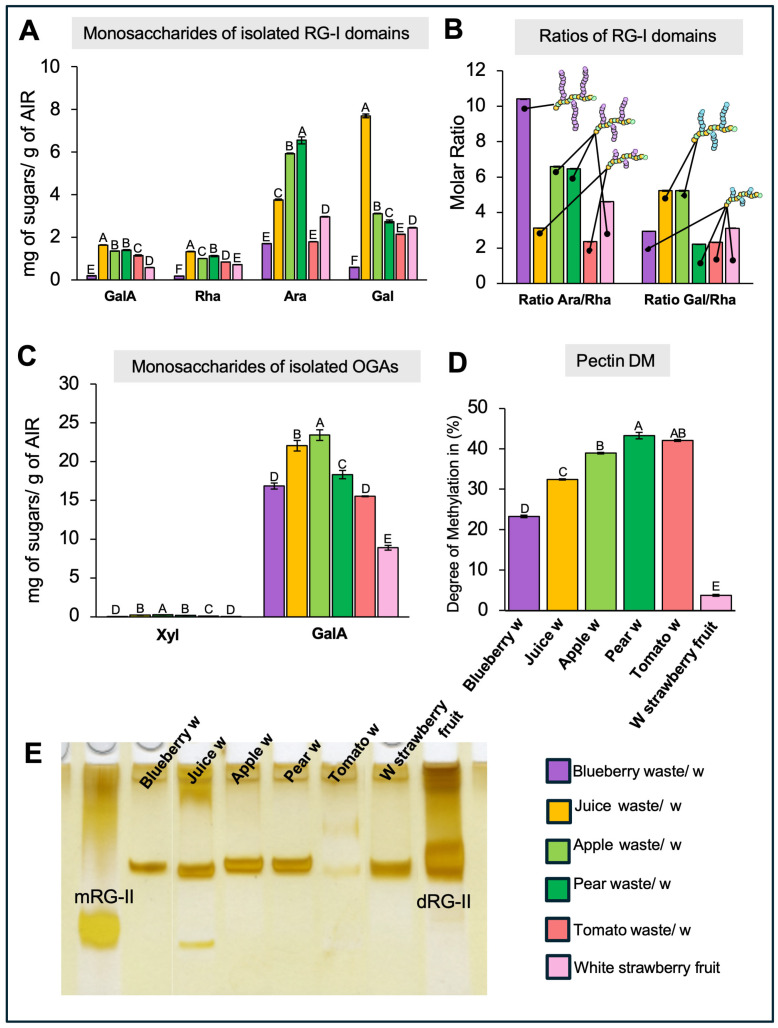
Analysis of Different Pectic Domains. (**A**) Monosaccharide composition of isolated RG-I domains from selected fruit samples, including blueberry, juice, apple, pear, tomato wastes (/w), and fresh white strawberry (W strawberry fruit). The main components of the RG-I backbone, galacturonic acid (GalA), and rhamnose (Rha), are shown alongside arabinose (Ara) and galactose (Gal), which make up the lateral chains of RG-I (*n* = 3). The data highlight varying proportions of these monosaccharides, underscoring the structural diversity of RG-I across different fruit sources. Bars indicate standard deviation (SD). (**B**) Ratios derived from the data in panel (**A**), illustrating the degree of branching in RG-I across the different fruit waste samples (*n* = 3). The results reveal distinct branching characteristics, indicating variability in RG-I architecture among the analyzed samples. (**C**). Monosaccharide composition of isolated oligogalacturonides (OGAs) from the same fruit waste samples, focusing on GalA and xylose (Xyl), key components of homogalacturonan (HG) and xylogalacturonan (XGA) domains (*n* = 3). The composition varies significantly among samples, suggesting distinct structural profiles of pectic regions in each fruit source. Bars represent standard deviation (SD). (**D**) Degree of methylesterification of pectins extracted from each fruit waste sample (*n* = 15), indicating the extent of methylation within the pectic chains. Bars represent standard error (SE). (**E**) Gel electrophoresis image showing the presence of rhamnogalacturonan-II (RG-II) structures in the analyzed samples, with bands corresponding to RG-II monomers (mRG-II) and dimers (dRG-II) labeled. Samples are presented left to right: blueberry, juice, apple, pear, tomato waste, and fresh white strawberry (*n* = 3). All samples contain RG-II dimers with varying abundance in their cell wall matrices. Statistical analysis was performed using one-way ANOVA followed by Tukey’s post hoc test (*p* < 0.05).

The monosaccharide composition analysis of AIR or enriched pectin- and HC-fractions provides insight into overall differences among the samples; however, it does not offer detailed information about the specific structures within the various pectin and HC domains. To address this, specific pectin domains were isolated by an initial treatment with endo-PG followed by size-exclusion chromatographic fractionation [30,31]. In this way, the RG-I and oligogalacturonan (OGA) domains were separated, collected, pooled, and then their sugar compositions were analyzed by HPLC after TFA hydrolysis (Figure 3). The RG-I domains isolated from blueberry, juice, apple, pear, tomato, and white strawberry display varying amounts of key sugars, including GalA, Rha, Gal, and Ara (Figure 3A). Interestingly, the GalA/Rha molar ratio for all RG-I samples is close to 1, confirming the structure of the RG-I backbone. Juice waste harbors the highest content of both Rha and GalA, followed by pear and apple, while lower levels are observed in tomato, white strawberry, and blueberry. The presence of Gal and Ara in varying proportions across the samples suggests the formation of branched side chains attached to the RG-I backbone, indicating a high degree of structural complexity. As observed in Figure 3A, the RG-I domain isolated from pear is particularly rich in Gal, suggesting a predominance of galactan or arabinogalactan chains rather than arabinan branches (Figure 3A). In contrast, apple and juice residues are notably enriched in Ara relative to Gal, indicating a greater abundance of arabinan side chains. To estimate the degree of branching within RG-I domains, the molar ratios of Ara/Rha and Gal/Rha were calculated for each sample (Figure 3B). Blueberry, pear, and apple exhibited the highest Ara/Rha ratios (10.4, 6.6, and 6.5, respectively), suggesting a higher prevalence of arabinan branches. Conversely, juice and apple RG-I domains showed the highest Gal/Rha ratios (5.2 for both), indicating a significant presence of galactan-rich side chains.

**Figure 4:**
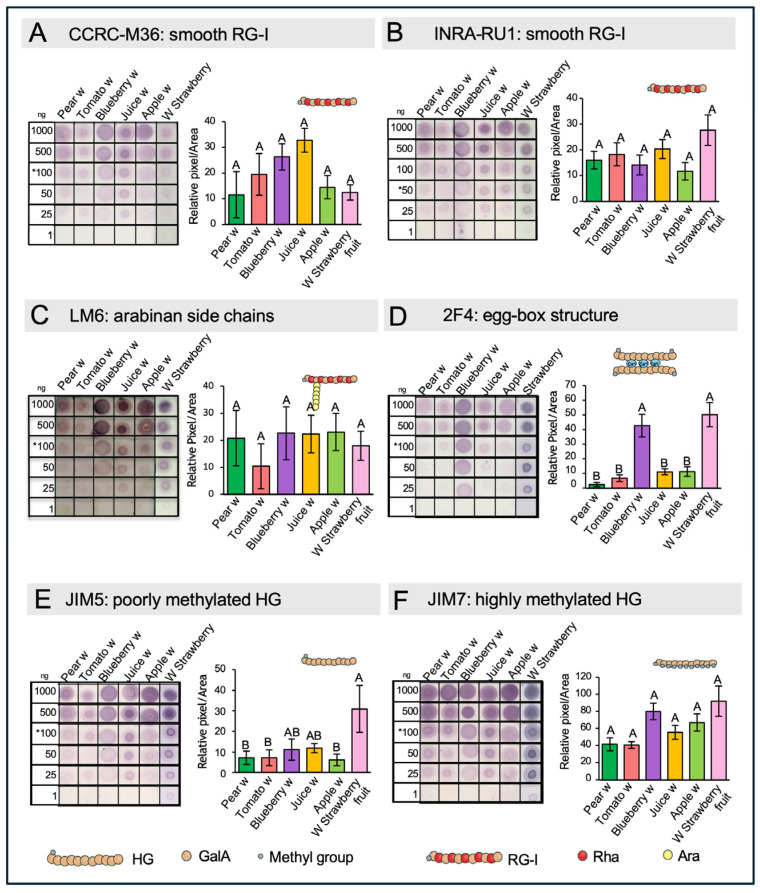
Immunodetection of Specific Pectin Domains in Pectin-Enriched Fractions. (**A**) Detection of smooth RG-I with CCRC-M36 antibody. (**B**) Detection of smooth RG-I with INRA-RU1. (**C**) Detection of smooth Arabinan side chains with LM6 antibody. (**D**–**F**) Detection of HG with different level of methylation with 2F4, JIM5, and JIM7 antibodies. Dot blot assays were performed using pectin-enriched fractions extracted with imidazole and ammonium oxalate, applied at different dilutions. Each antibody’s target epitope is illustrated by a cartoon above its corresponding graph. Quantification was carried out at the dilution marked with an asterisk. Different letters indicate statistically significant differences between samples. Standard error (SE) was calculated from three to five technical replicates. Statistical analysis was performed using one-way ANOVA followed by Tukey’s post hoc test (*p* < 0.05).

To verify the presence of RG-I epitopes within the intact pectic matrix, a dot blot analysis was performed using specific antibodies (Figure 4A–C). The antibodies INRA-RU1 and CCRC-M36 were used to detect the unbranched backbone of RG-I, while LM6 targeted the arabinan side chains. Unbranched RG-I was detected in all samples, with no significant differences in relative abundance among them (Figure 4A,B). Similarly, detection of arabinan side chains showed no significant variation across samples (Figure 4C).

### 2.5. Analysis of the Isolated Oligogalacturonans

The analysis of isolated OGA domains (Figure 3 C,D) reveals variations in GalA content among the different waste types. Juice and apple waste samples exhibit higher GalA levels, indicating a strong presence of HG domains in these samples. Traces of Xyl are detected in all samples, suggesting the presence of xylogalacturonans (XGA), a Xyl-branched form of HG, albeit in low amounts. As HG can display different degrees of methylesterification (DM), this parameter was measured as described in Sanhueza et al. [30,31] (Figure 3D). White strawberry exhibits a low DM (3.7%) along with a minimal Xyl content (approximately 0.02 mg g^−1^), indicating that HG in this fruit exists primarily in a demethylesterified form with low substitution levels. In comparison, other pectin-rich wastes such as blueberry, apple, pear, tomato, and juice residues show higher DM values, ranging between 23.2% and 42.07%, with slightly more Xyl (though still below 0.25 mg g^−1^ of pectin). Papaya mucilage contains highly methylesterified HG (~85%), as reported by Sanhueza et al. [30].

To confirm the variations in the HG pectin domains between the wastes, dot blot assays against different pectin epitopes were performed on isolated pectin fractions (Figure 4D–F). To evaluate HG epitopes, the antibodies 2F4, JIM5, and JIM7 were used to recognize the egg-box structure, as well as poorly and highly methyl-esterified forms of HG, respectively. The results show increased detection of the egg-box structure and poorly methyl-esterified HG in blueberry and white strawberry samples, confirming their lower DM (Figure 4D,E). JIM7 labeling was intense in all samples, although no significant differences between them were recorded (Figure 4F), indicating that these samples also contain highly methyl-esterified HG.

### 2.6. Rhamnogalacturonan-II Detection and Quantification

To evaluate the existence of RG-II in the waste samples, AIR was treated with endo-PG, and RG-II domains were visualized by PAGE, enabling the identification of both monomeric and dimeric forms. The results, shown in Figure 3E, highlight the presence of RG-II monomers (mRG-II) and dimers (dRG-II) in the fruit waste samples, with varying intensities. Notably, all analyzed samples, except for tomato waste, which shows only trace amounts, contain RG-II dimers. Interestingly, juice waste also exhibits mRG-II, indicating variability in the degree of RG-II dimerization among the samples.

### 2.7. Dot Blot Assays of Hemicellulose

**Figure 5:**
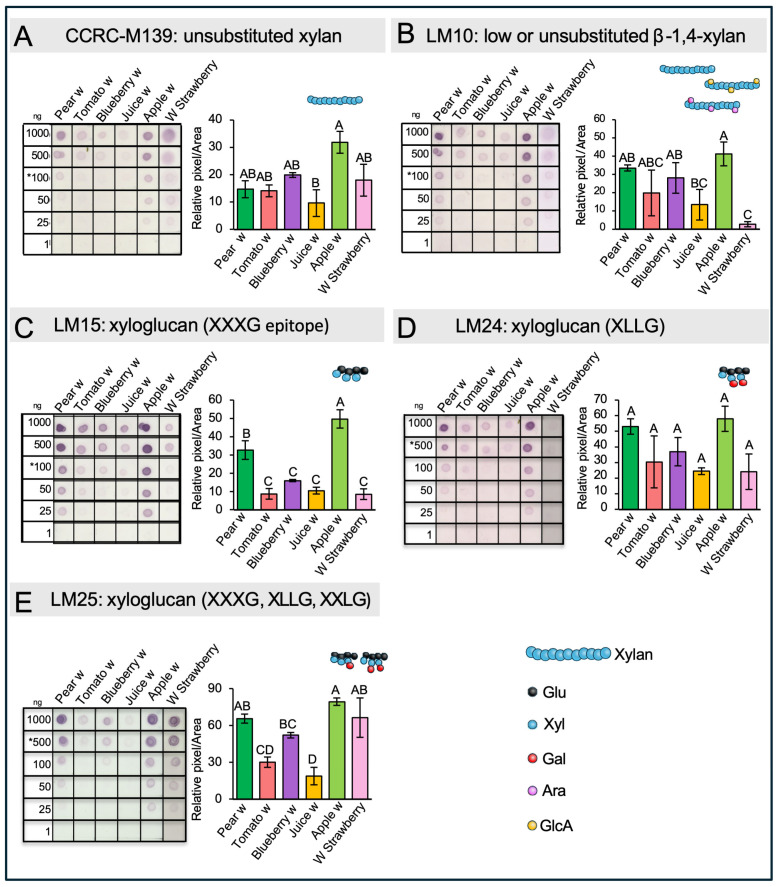
Immunodetection of Specific Hemicellulose Domains in Hemicellulose-Enriched Fractions. (**A,B**). Detection of unsubstituted xylans with CCRC-M139 and LM10 antibodies. (**C**–**E**) Detection of xyloglucans with LM15, LM24 and LM25 antibodies. The epitope recognized by each antibody is illustrated with a schematic cartoon above each corresponding graph. Dot blot assays were performed using serial dilutions of hemicellulose-enriched fractions, with antibodies targeting specific hemicellulose structures. Quantification was conducted at the dilution marked with an asterisk. Different letters indicate statistically significant differences between samples (*p* < 0.05), based on one-way ANOVA followed by Tukey’s post hoc test. Error bars represent the standard error (SE) from three to five technical replicates.

To gain information about the structure of HC and to determine the specific types present in the waste materials, immunolabeling assays after transferring chemically isolated HCs onto nitrocellulose membranes were performed. We used specific antibodies to detect xylan and xyloglucans. To distinguish between the substituted and unbranched forms of xylan, two antibodies were employed: CCRC-M139, which recognizes an unsubstituted pentamer of beta-(1,4)-Xyl, and LM10, which can recognize unsubstituted and relatively low-substituted xylans (Figure 5A,B). The results reveal the presence of both forms of xylan in all the waste materials, with higher detection levels observed in apple and pear residues. In tomato, blueberry, and juice, there was a similar low detection signal with both antibodies directed to substituted or unbranched xylan. Interestingly, CCRC-M139 shows stronger signals in white strawberries, while the detection of low-substituted or unsubstituted xylan was comparatively weak in this material.

To identify different forms of xyloglucan in the waste materials, the antibodies LM15, LM24, and LM25 were employed (Figure 5C–E). These antibodies specifically recognize various xyloglucan motifs: LM15 detects XXXG, LM24 binds to XLLG, and LM25 targets XXXG, XXLG, and XLLG. All three epitopes were detected across the waste samples, although at different intensities, and the highest intensity was recorded in pear and apple residues.

## 3. Discussion

### 3.1. Analysis of Fruit Waste Samples Revealed a High Carbohydrate Content with Diverse Polysaccharide Compositions

One advantage of this agro-industrial waste material is that, since it originates from food production, one of the key requirements is the stability of the final product offered to consumers. In this sense, the composition of the different analyzed pulps should be relatively consistent. Given that these waste materials are generated in large quantities, they represent a promising source of various cell wall polymers. With this in mind, one of the main points highlighted in the present work is the high yield and diversity of monosaccharides—and consequently, of polymeric structures—found in the different samples analyzed. The extraction yield of cell wall material from fruit wastes is significantly higher compared to fresh fruit, as most of the water (95–99% in fruit) is extracted during the manufacture of pastes, pulps, and juices, and the residue that remains is made up of insoluble fats and fibers (Supplementary Table S1). The high levels of Xyl in blueberry and pear waste measured in AIR suggest an abundance of HCs, such as xyloglucan, xylan, or arabinoxylans. This corresponds with a higher yield of AIR, indicating a dense and structured cell wall matrix. The elevated Glc in fruit juice waste suggests a predominance of xyloglucans or cellulose. The GalA richness in papaya aligns with previous findings that reported homogalacturonans (HG) as the main component of papaya mucilage [30]. The presence of approximately 30% GalA in tomato, apple, and white strawberry suggests a substantial presence of acidic pectins, which could include HG, xylogalacturonan (XGA), rhamnogalacturonan I (RG-I), and/or RG-II. The wide variation in Ara and Gal content is important to consider, as these sugars are found in both pectins and HCs but are primarily associated with RG-I branching and arabinogalactan proteins. These differences highlight the potential of repurposing various fruit wastes as valuable sources of polysaccharides, including pectins, cellulose, and hemicelluloses.

The differences in AIR yield, pectin, and HC content among the fruit wastes reflect the distinct composition and structural roles of these polysaccharides in the cell wall. Importantly, fruit wastes obtained from our Agro-industry partner (Agrozzi) have been previously treated with pectinases during their industrial process, and this could explain the lower level of pectins identified in these agro-industrial samples. For example, apple and pear wastes, with their high HC content, suggest a denser AIR structure, which may explain the high carbohydrate extraction yields compared to the other fruit sources. However, this is not the case for blueberry waste, where similar amounts of pectin and HC are extracted despite a higher AIR yield. This suggests that beyond polysaccharide proportions, the structure and organization of these molecules influence cell wall isolation and extractability. Tomato and white strawberry, which have lower AIR yields but moderate pectin and HC content, point to more flexible cell wall structures. Examining the differences in pectin and HC extraction highlights the potential of fruit waste as a valuable source of polysaccharides for industrial applications. In particular, pear, apple, and white strawberry stand out as promising candidates for HC extraction. Meanwhile, white strawberry, juice, and blueberry waste may offer strong potential for pectin-based products. Papaya fruit waste is especially promising for both HC and pectin extraction due to its hydrophilic mucilage-like nature [30], which facilitates the accessibility of various carbohydrates.

### 3.2. Chemical Analysis of Pectins- and HC-Enriched Fractions Suggest Variations in Pectins and Hemicellulose Compositions

The pectin GalA content in the pectin fractions aligns with expectations, as GalA is the main component of homogalacturonan and is also found within the RG-I domain. The variation in GalA levels among the samples—particularly the high content in white strawberry and low in juice waste—reflects differences in both the abundance and composition of pectins, especially in the relative proportions of HG and RG-I, the two main pectic domains. For example, papaya mucilage contains 96.6 mg g^−1^ of GalA [30], while blueberry cultivars range from 120 to 350 mg g^−1^, depending on ripening stage [31]. These variations indicate that different fruit wastes contain distinct pectin domains.

Despite the low Rha content, all samples appear to contain some level of RG-I domain. However, RG-I is highly variable due to its branching, which is key to understanding its structure and potential functional properties. A useful way to infer the degree of RG-I branching is through Ara and Gal content. The high Ara and Gal levels in white strawberry, pear, apple, and juice waste suggest highly branched RG-I structures in these samples. However, it remains unclear whether these side chains are shorter and longer, or shorter but more densely distributed along the RG-I backbone. The observed differences in Ara and Gal content indicate diverse RG-I architectures, underlining the importance of further structural analysis.

The HC analysis focused on Glc, Xyl, and Gal, as the first two are the main sugars in xyloglucan—the predominant hemicellulose in dicotyledons—while Gal is one of the sugars that can be attached to Xyl in the side chains. The variability in composition across samples, particularly the low HC sugar content in blueberry waste and the high values in juice and apple, indicates differences in hemicellulose abundance and structure, likely reflecting both species-specific characteristics and processing effects. Notably, the lower Gal proportion in the HC fractions of pear and tomato HC suggests possible differences in the xyloglucan side chain composition, which may affect the physicochemical properties of the hemicellulose fraction.

### 3.3. The Amount and Structure of Rhamnogalacturonan-I (RG-I) Domains Vary Across Different Waste Types

The monosaccharide composition analysis of the isolated RG-I domain provides several insights. First, it confirms the presence of the RG-I domain in all samples, although with varying abundance. Additionally, it offers information about the degree and type of branching—for example, whether arabinan or galactan is the predominant side chain—and potentially about the length or density of these side chains along the RG-I backbone. In the case of blueberry, we observed low levels of GalA and Rha, suggesting a limited presence of the RG-I backbone. However, the Ara/Rha molar ratio was the highest among all samples, strongly indicating that the RG-I present in blueberry is enriched in long arabinan side chains. In contrast, the juice sample showed the highest amounts of Rha and GalA, but one of the lowest Ara/Rha ratios. This suggests that the arabinan side chains are either shorter or that the Ara residues are more likely part of arabinogalactan side chains rather than pure arabinan. Additionally, it is not possible to discard that the detected arabinan or galactan signals could originate from arabinogalactan proteins, as previously reported by Sanhueza et al. [31].

The analysis of RG-I domains using specific antibodies aims to provide insights into the actual structures present within the pectin matrix. In this context, it is important to consider that detection and/or epitope accessibility issues may arise, particularly because the dot blot assays were performed on the entire pectin matrix rather than on isolated RG-I domains. Another limitation relates to antibody epitope specificity. For example, CCRC-M36 and INRA-RU1 do not yield the same detection pattern, despite both recognizing unbranched RG-I. This discrepancy is due to the number of Rha-GalA disaccharide repeats each antibody targets—three for CCRC-M36 and six for INRA-RU1. The use of LM6 confirmed the presence of arabinan chains in all samples, although with varying levels of abundance. Overall, the results are consistent with the observed Ara/Rha molar ratios, except in the juice and pear samples. These discrepancies may be explained by the use of whole pectin extracts instead of isolated domains, which could lead to steric hindrance and affect antibody accessibility.

Nonetheless, by combining monosaccharide compositional analysis of isolated RG-I domains with dot blot results, we confirm the presence of RG-I structures and highlight their structural diversity across different fruit waste sources. This variability underscores the potential of these fruit residues for targeted applications, such as the use of arabinan- or galactan-rich RG-I fractions in specific industrial processes.

### 3.4. The Isolation of OGAs Revealed Variations in HG Quantity and Degree of Methylation, as Well as the Presence of Xylogalacturonan Across Different Types of Waste

A remarkable difference in the calculated degree of methylesterification (DM) was observed between fresh white strawberry and the various industrial waste samples analyzed. This finding suggests that these discarded materials could serve as valuable sources of highly methyl-esterified pectins—an attribute that can be modulated to tailor gelling properties for specific applications. This parameter is particularly relevant in the food industry, where pectins are widely used as thickeners. The strong recognition by the JIM7 antibody observed in white strawberry may seem unexpected, given the low methylation levels detected. However, DM reflects only the overall abundance of methyl groups on GalA residues and does not account for their spatial distribution. In this case, the pattern of methylation may allow for antibody recognition despite the low overall DM. Something similar was observed with blueberry when analyzed using the 2F4 antibody. Despite having a much higher DM than white strawberry, it exhibited a similar abundance of egg-box structures—formed when long stretches of HG are de-methylesterified. This supports the idea that the DM reflects the overall methanol content but does not provide information about its distribution along the polymer chain. Taken together, these results indicate that DM varies among fruit waste samples, a key characteristic that plays a crucial role in determining their pectin gelling behavior.

### 3.5. RG-II Detection and Quantification in Fruit Waste Samples Reveal Variations in Amount and Structure

The findings presented here suggest that most of the tested residues could serve as viable sources of RG-II, with the exception of tomato waste, which has a low accumulation of RG-II in its cell walls. The variability in RG-II content also implies that these residues could be selectively processed to generate RG-II-enriched materials for diverse applications, such as biomedical uses.

### 3.6. Dot Blot Assays of Hemicellulose Fractions Reveal the Presence of Xyloglucans and Xylan in the Different Wastes

The results obtained using xylan-specific antibodies revealed the presence of this domain in both unsubstituted and low-substituted forms. A noteworthy outcome from the dot blot analyses is the clear variation observed among the samples, which is significant when assessing polymeric diversity. In the case of substituted xylans, we refer to heteroxylans—a family of polysaccharides with a xylose backbone that can be substituted with arabinose (Ara) or glucuronic acid (GlcA), resulting in arabinoxylans and glucuronoxylans, respectively. Among the analyzed samples, pear and apple showed strong signals with both antibodies and also exhibited a remarkably high Ara content (Supplementary Table S2), suggesting that the detected polymer is likely arabinoxylan.

The use of antibodies targeting different xyloglucan epitopes not only confirmed their presence but also provided insights into structural variations among the samples. LM15, which recognizes the basic form of xyloglucan (XXXG), revealed that this domain was present in all samples, albeit with varying abundance. LM24, which specifically detects the XLLG motif—characterized by two Gal residues attached to distinct Xyl units—showed this epitope in five out of six samples, with particularly strong signals in pear and apple. This correlates with the high Gal content found in their respective HC fractions (Figure 2B Supplementary Table S2). In contrast, strawberry exhibited a negligible signal with LM24. Interestingly, the response of the strawberry to LM25 was the opposite, suggesting that this sample likely contains XXLG as part of its xyloglucan structure.

## 4. Methodology

### 4.1. Plant Residue Material

Agro-industrial wastes, corresponding to blueberry (*Vaccinium corymbosum* L.), pear (*Pyrus communis* L.), apple (*Malus domestica* L.), and tomato (*Solanum lycopersicum* L.), had been subjected to traditional industrial processing in order to obtain fruit sauces, jams, and pastes. The discarded material corresponds to pomaces. The processing was performed by Agrozzi, a local agroindustry company located in Teno (Curicó, Maule Region, Chile), during March–April 2022. Fruit juice samples correspond to a concentrated fruit syrup, also prepared by Agrozzi. In the case of Chilean papaya (*Vasconcellea pubescens*), the sample corresponds to discarded material (seed mucilage) obtained from a mini processing factory located in Licantén (Curicó, Maule Region, Chile), during the preparation of papaya preserves and jams. Additionally, in the case of white Chilean strawberry (*Fragaria chiloensis* (L.) Mill), the sample corresponds to a mixture of fruit at different developing stages (C1 to C4 stages as reported by Mattus-Araya et al. [32]. Strawberry fruit was collected during December 2021 from a commercial field (Purén, Araucania Region, Chile) and transported immediately to the laboratory. Once obtained, the different fruit/waste samples were frozen under liquid nitrogen and stored at −20 °C before processing.

### 4.2. AIR Preparation

To prepare the alcohol-insoluble residue (AIR), the fruit/waste samples were ground in liquid nitrogen and subjected to two extractions. The first involved an overnight incubation followed by an eight-hour incubation in 80% ethanol with agitation at room temperature (RT). Lipids were then removed by rinsing the pellet twice for 2 h with a mixture of methanol:chloroform (1:1) and twice for 1 h with acetone. Each time, the supernatant was removed by centrifugation at 3000× *g* for 5 min. The final AIR was dried overnight at RT and weighed.

### 4.3. Cell Wall Fractionation

Different cell wall-enriched fractions were obtained from the AIR through sequential chemical extraction. Pectins were extracted by incubating the AIR with 0.5 M imidazole (pH 7.0) at RT, followed by two incubations with 0.2 M ammonium oxalate at 60 °C. Centrifugations at 3000× *g* for 5 min were performed after each incubation, and the resulting supernatants were dialyzed against water. After dialysis, the pectic fractions obtained with imidazole and ammonium oxalate were combined into a single pectic fraction prior to freeze drying. The remaining pellet from pectin extraction was used to isolate hemicelluloses by rinsing it with water and then incubating it overnight at 37 °C on a shaker with 6 M NaOH/1% NaBH_4_ followed by a centrifugation step. This process was repeated twice, and the collected supernatants were dialyzed against water and freeze-dried.

### 4.4. Acid Hydrolysis of Cell Wall Material (AIR, Pectin-, and Hemicellulose-Enriched Fractions) and Monosaccharide Quantification by HPAEC-PAD

Samples underwent hydrolysis for 1 h using 400 μL of 2 M trifluoroacetic acid (TFA) at 121 °C. TFA was then evaporated at 45 °C using nitrogen, and the samples were rinsed twice with 400 μL of 100% isopropanol. The sample was resuspended in 600 μL of MilliQ water, sonicated for 15 min, centrifuged for 1 min at 12,100× *g*, and finally filtered through a syringe with a pore size of 0.22 μm. It was then transferred to a new tube and analyzed using HPAEC-PAD. Inositol and allose (250 µM each) were utilized as internal controls for TFA hydrolysis.

Monosaccharide quantification was performed using a Dionex ICS3000 (Dionex Corporation, Sunnyvale, CA, USA) ion chromatography system equipped with a Pulsed Amperometric Detector, a CarboPac PA1 (4 × 250 mm) analytical column, and a CarboPac PA1 (4 × 50 mm) guard column. For the separation of neutral sugars, the system was set at 32 °C with a flow rate of 1 mL min^–1^ using a 20 mM NaOH isocratic gradient for 24 min. Subsequently, for the separation of acidic sugars, a solution of 75 mM sodium acetate and 150 mM NaOH was employed for 20 min at a flow rate of 1 mL min^–1^ at 32 °C. A final wash step using 200 mM NaOH for 5 min was performed after each run, followed by column equilibration with 20 mM NaOH for 6 min. Quantification relied on standard curves established for both neutral sugars [fucose (Fuc), rhamnose (Rha), arabinose (Ara), galactose (Gal), glucose (Glc), xylose (Xyl) and mannose (Man)], and acidic sugars [galacturonic acid (GalA) and glucuronic acid (GlcA)].

### 4.5. Methylesterification Analysis

The degree of methylesterification (DM) was determined in pectin preparations, following the method of Anthon and Barrett [33]. Pectin samples were resuspended in MilliQ water to a concentration of 3 mg mL^−1^, and 50 μL of each sample solution was saponified with 50 μL of 0.2 M NaOH and incubated for 1 h at 4 °C on ice. Afterward, 50 μL of 0.2 M HCl was added to stop the saponification of pectin samples. Then 150 µL of MilliQ water was added to reach a total volume of 300 µL. To 50 μL of the final saponification mixture, 100 μL of 200 mM Tris-HCl (pH 7.5), 40 μL of 3 mg mL^−1^ 3-methyl-2-benzothiazolinone hydrazone (MBTH), and 20 μL of 0.02 U μL^−1^ alcohol oxidase from *Pichia pastoris* (Sigma-Aldrich, Saint Louis, MO, USA) were added. The mixture was incubated for 20 min at 30 °C. To determine the methanol content, 200 µL of sulfamic acid and ammonium ferric sulfate dodecahydrate (0.5% *w*/*v* each in water) were added to the previous mixture and incubated at RT for 20 min. Finally, 600 µL of MilliQ water was added, and the absorbance at 620 nm was measured. The methanol content was determined using a standard curve between 0 and 10 µg µL^−1^ of methanol. All determinations were performed with five technical replicates.

### 4.6. Uronic Acid Quantification

The quantification of uronic acids in pectin fractions was performed using the m-hydroxybiphenyl method, following the protocol outlined by Blumenkrantz and Asboe-Hansen [34]. This involved mixing 2 µL of a 3 mg mL^−1^ pectin solution with 18 µL of water and 100 µL of a 0.5% solution of N_2_B_4_O_7_·10H_2_O (borax) in sulfuric acid. The samples were incubated at 100 °C for 5 min, and absorbance was recorded at 520 nm. Color development was initiated by adding 2 µL of 0.15% m-hydroxybiphenyl in 1 M NaOH solution, followed by measuring the absorbance at 520 nm after standing for 5 min at RT. Uronic acid content was determined using a standard curve based on 0.1 to 2 µg of GalA. All measurements were performed using six technical replicates.

### 4.7. Pectin Domain Isolation

The AIR was saponified by incubation with 1 M Na_2_CO_3_ overnight at 4 °C, followed by neutralization with acetic acid and two rinses with water. The saponified AIR was then digested overnight at RT with 5 U mL^−1^ of endopolygalacturonase (endo-PG) from *Aspergillus aculeatus* (Megazyme, Wirclow, Irland) in pyridine:acetic acid:water (PyAW 1:10:200, *v*/*v*/*v*). The digestion was loaded onto a BioGel P-30 column (2.5 × 57 cm) and eluted with PyAW 1:1:98 (*v*/*v*/*v*) at 1 mL min^−1^. To detect the fractions where the different domains eluted, 20 µL from each fraction was used to quantify total uronic acids. Fractions enriched in RG-I, RG-II, and oligogalacturonides (OGAs) were separately pooled and dried in a speed vacuum.

### 4.8. Electrophoretic Analysis of RG-II

Fractions collected from the BioGel P-30 column and corresponding to RG-II were pooled and concentrated using a speed vacuum and then reconstituted in water. The content of uronic acids was quantified, and then RG-II dimerization status was analyzed by PAGE as described in Sanhueza et al. [35]. For each sample, 0.8 µg was loaded onto a 26.4% acrylamide gel. As weight control markers, 4 µL of RG-II monomer or RG-II dimer (1.8 µg µL^−1^) were used. Briefly, the electrophoresis was conducted for 75 min at 200 V, and the gel was subjected to a silver staining procedure [36].

### 4.9. Immunodot Blot Assay

Immunodot blot assays were conducted utilizing purified pectin and HC fractions obtained from the AIR. The primary antibodies employed are listed in the Supplementary Table. Serial dilutions were prepared from the purified pectin and HC fractions, commencing with an initial amount of 1000 ng. Subsequently, 1 µL from each dilution was blotted onto a 0.45 µm nitrocellulose membrane (Thermo Scientific, Rockford, IL, USA). These blotted membranes were incubated with a blocking solution containing 2% *w*/*v* of powdered skimmed milk dissolved in TBS (25 mM Tris, 0.15 M NaCl) supplemented with 0.1% *v*/*v* of Tween-20 (TBS-T). All primary antibodies were diluted to 1:50 in TBS-T with 1% *w*/*v* skimmed milk, and the membranes were incubated for 2 h at RT.

Following incubation with the primary antibody, the membranes underwent three washes with TBS-T and were then incubated for 1.5 h at RT with the appropriate secondary antibody (Supplementary Table S4), which was diluted to 1:2000 in TBS-T. Finally, the membranes were washed and incubated with 1 Step BCIP/NBT (5-bromo-4-chloro-3-indolyl-phosphate/nitro blue tetrazolium) chromogenic alkaline phosphatase (AP) substrate developing solution (Thermo Scientific, Rockford, IL, USA). The reaction was terminated after three washes with distilled water. The dot signals from pectin and HC fractions were semi-quantified using ImageJ 1.53 software (Freeware, National Institute of Health). A minimum of three technical replicates were performed for each pectin and HC fraction.

### 4.10. Statistical Analysis

One-way ANOVA analyses were conducted with the Tukey test (significance level of 0.05).

## 5. Conclusions

The analysis of polysaccharide composition, extraction yields, and structural variations across six agro-food fruit waste samples—blueberry, juice, apple, pear, tomato, and papaya—and white strawberry as a non-agro-food reference, highlights their potential as sustainable sources of industrially valuable polysaccharides. The structural diversity of pectins and hemicelluloses among these samples reflects significant variability in cell wall complexity, which can be harnessed for various applications.

Polysaccharides offer distinct rheological and mechanical properties, making them suitable for a wide range of industrial uses, including food, cosmetic, and biomedical products. Detailed compositional insights enable the development of tailored applications, adding value to waste materials and supporting sustainable circular practices in the agro-food sector.

Among the samples, blueberry, pear, and apple stood out as rich sources of hemicelluloses, while juice, papaya, and white strawberry showed high potential for pectin-based applications. Notably, papaya mucilage demonstrates versatility for both HC and pectin extraction due to its hydrophilic nature [30].

The detection of RG-I and RG-II pectin domains, particularly in juice and white strawberry, suggests potential for functional ingredient development, as RG-I has been associated with anti-tumor, anti-diabetic, gastroprotective, and neuroprotective properties [37,38]. Additionally, variations in the degree of methylesterification (DM) influence pectin gelling behavior, relevant for soft or firm gel formulations in food and cosmetics [39].

High levels of xylans and xyloglucans in pear and apple waste, along with unique substitution patterns, further support their potential use as dietary fiber additives in the agro-food industry.

These findings underscore the importance of developing eco-friendly extraction protocols that align with industrial sustainability goals. By integrating targeted cell wall analyses with green extraction methods, industries can recover specific polysaccharides efficiently, transforming waste into high-value materials and promoting more sustainable production chains.

## Data Availability

All data generated or analyzed during this study are included in this article and/or in its Supporting Information.

## Supplementary Materials

The following supporting information can be downloaded at: https://www.mdpi.com/article/10.3390/ijms26104942/s1. References [40,41,42,43,44,45,46,47,48] are cited in the supplementary materials.
