# Supplementary material for: Back to the Wastes: The Potential of Agri-Food Residues for Extracting Valuable Plant Cell Wall Polysaccharides
Source: Int J Mol Sci. 2025 May 21;26(10):4942. doi: 10.3390/ijms26104942 (PMC12112314; doi:10.3390/ijms26104942)
Supplement: Supplementary file 1 [file ijms-26-04942-s001.zip › ijms-3611596 Table S1.pdf]

Table S1. Extraction of cell wall polysaccharides from each type of fruit source. The yields foor the AIR were calculated based on the AIR extracted, divided by the fresh sample weight. Fresh white strawberry fruit was used as a control to compare the polysaccharide content of the residues. Yield of pectin and hemicellulose enriched fractions extracted from AIR residues prepared from fruit waste samples. Yields were expressed in percentage calculated by dividing the amount of pectin or hemicellulose extracted by the AIR content.

Sample	Fresh weight (g)	AIR (g)	AIR yield (%)	Pectin yield (%)	HC yield (%)
Blueberry waste	41.03	21.17	51.6	17.3	15.6
Fruit juice waste	32.46	4.95	15.2	19.5	17.0
Apple waste	41.12	8.14	19.8	10.3	21.3
Pear waste	43.75	11.45	26.2	9.0	30.7
Tomato waste	31.26	5.77	18.5	13.1	13.7
Papaya mucilage discard	23.30	0.27	1.2	40.5	23.2
White strawberry fruit	50.06	1.27	2.5	30.3	19.8
