# Supplementary material for: Back to the Wastes: The Potential of Agri-Food Residues for Extracting Valuable Plant Cell Wall Polysaccharides
Source: Int J Mol Sci. 2025 May 21;26(10):4942. doi: 10.3390/ijms26104942 (PMC12112314; doi:10.3390/ijms26104942)
Supplement: Supplementary file 1 [file ijms-26-04942-s001.zip › ijms-3611596 Table S2.pdf]

Table S2. Monosaccharide quantification for AIR, pectins, hemicelluloses, RG-I isolated domain and OGAs.

	Fucose		Rhamnose		Arabinose		Galactose		Glucose		Mannose		Xylose		GalA		GlcA	
AIR	mg monosaccharide / g AIR																	
**Blueberry**	0.47	(0.04)	3.89	(0.12)	13.99	(0.20)	20.47	(0.42)	13.70	(0.80)	4.06	(0.40)	140.33	(0.80)	21.44	(0.22)	0.95	(0.20)
**Juice**	0.43	(0.06)	9.60	(0.13)	26.53	(0.14)	79.87	(1.15)	83.82	(5.37)	29.82	(0.71)	35.06	(0.94)	70.69	(1.73)	4.50	(0.09)
**Apple**	7.05	(0.25)	13.48	(0.54)	64.64	(0.67)	48.85	(1.99)	23.45	(1.21)	8.87	(0.03)	58.87	(4.02)	84.98	(2.95)	1.91	(0.02)
**Pear**	3.97	(0.13)	10.68	(0.28)	52.20	(1.99)	27.29	(0.78)	14.62	(0.10)	8.32	(0.11)	119.89	(4.52)	63.27	(1.55)	2.23	(0.04)
**Tomato**	0.31	(0.01)	5.63	(0.06)	17.60	(0.86)	23.16	(0.55)	14.04	(0.51)	60.51	(1.97)	23.82	(1.20)	58.27	(2.80)	2.58	(0.04)
**White strawberry**	3.98	(0.22)	22.92	(0.73)	88.83	(4.50)	79.24	(4.24)	40.64	(1.63)	9.85	(0.74)	106.63	(13.26)	197.79	(11.92)	3.14	(0.15)
Pectins	mg monosaccharide / g pectin																	
**Blueberry**	0.71	(0.02)	7.53	(0.19)	32.52	(1.13)	30.54	(0.78)	15.68	(0.50)	4.34	(0.12)	32.32	(4.97)	188.36	(8.42)	2.80	(0.18)
**Juice**	0.51	(0.12)	11.65	(0.80)	27.31	(2.31)	69.23	(6.85)	28.93	(1.81)	5.76	(0.47)	8.44	(0.44)	131.28	(6.56)	3.69	(0.06)
**Apple**	2.38	(0.16)	10.97	(0.88)	58.13	(3.85)	28.61	(2.05)	20.66	(1.29)	1.22	(0.15)	16.98	(1.20)	235.88	(18.23)	3.54	(0.24)
**Pear**	1.87	(0.01)	16.04	(0.11)	81.17	(0.61)	30.79	(0.20)	18.32	(0.16)	5.66	(0.12)	17.71	(0.19)	192.43	(2.77)	3.84	(0.08)
**Tomato**	0.38	(0.04)	8.12	(0.66)	17.23	(1.56)	23.15	(2.20)	6.40	(0.64)	4.33	(0.47)	5.83	(0.65)	182.18	(14.25)	3.53	(0.22)
**White strawberry**	N.D		13.70	(0.17)	69.50	(1.61)	48.06	(0.61)	17.44	(0.55)	1.78	(0.02)	13.44	(0.49)	289.20	(6.00)	N.D	
Hemicellulose	mg monosaccharide / g hemicellulose																	
**Blueberry**	1.28	(0.04)	7.37	(0.12)	50.31	(0.20)	62.57	(0.42)	89.49	(0.80)	16.94	(0.39)	88.17	(0.80)	9.10	(0.22)	0.92	(0.20)
**Juice**	0.29	(0.03)	14.31	(1.33)	50.46	(4.51)	125.64	(6.09)	136.37	(12.88)	23.24	(1.78)	135.80	(12.63)	31.86	(2.64)	14.26	(1.23)
**Apple**	24.12	(3.28)	65.41	(3.71)	253.73	(5.81)	162.60	(7.57)	151.31	(15.03)	9.07	(1.36)	176.18	(14.83)	121.18	(2.50)	6.24	(0.22)
**Pear**	13.23	(1.25)	23.48	(1.53)	127.91	(6.90)	63.86	(5.19)	107.14	(8.53)	10.71	(0.57)	117.64	(11.18)	50.24	(2.61)	5.83	(0.39)
**Tomato**	0.40	(0.02)	12.48	(0.39)	42.61	(0.21)	49.18	(0.36)	108.87	(1.24)	30.49	(1.13)	132.30	(1.60)	38.01	(1.65)	8.56	(0.31)
**White strawberry**	12.29	(2.22)	22.34	(2.67)	104.75	(14.36)	87.96	(13.12)	99.29	(17.19)	17.24	(2.21)	128.26	(19.77)	32.61	(3.62)	N.D	
OGAs	mg monosaccharide / g AIR																	
**Blueberry**	N.D		0.02	(0.001)	0.03	(0.001)	0.04	(0.002)	0.09	(0.006)	TR		0.04	(0.001)	16.86	(0.38)	N.D	
**Juice**	N.D		0.04	(0.002)	0.06	(0.002)	0.31	(0.007)	0.29	(0.006)	0.06	(0.002)	0.23	(0.01)	22.05	(0.68)	0.01	(0.001)
**Apple**	0.02	(0.001)	0.03	(0.001)	0.06	(0.003)	0.07	(0.004)	0.23	(0.01)	0.01	(0.003)	0.29	(0.02)	23.42	(0.69)	0.07	(0.01)
**Pear**	0.01	(0.000)	0.03	(0.001)	0.1	(0.003)	0.04	(0.002)	0.09	(0.003)	0.02	(0.001)	0.21	(0.01)	18.32	(0.54)	0.05	(0.004)
**Tomato**	N.D		0.02	(0.000)	0.02	(0.000)	0.04	(0.001)	0.07	(0.002)	0.01	(0.001)	0.10	(0.003)	15.53	(0.06)	0.02	(0.01)
**White strawberry**	N.D		TR		TR		0.05	(0.002)	0.23	(0.01)	N.D		0.02	(0.005)	8.90	(0.31)	N.D	
RG-I	mg monosaccharide / g AIR																	
**Blueberry**	0.01	(0.000)	0.18	(0.000)	1.69	(0.000)	0.59	(0.001)	0.07	(0.000)	N.D		0.05	(0.000)	0.19	(0.003)	TR	
**Juice**	0.06	(0.003)	1.33	(0.01)	3.76	(0.03)	7.69	(0.09)	0.56	(0.000)	N.D		0.16	(0.002)	1.63	(0.01)	0.02	(0.001)
**Apple**	0.03	(0.000)	1.00	(0.002)	5.93	(0.02)	3.11	(0.01)	0.18	(0.005)	N.D		1.44	(0.01)	1.35	(0.01)	0.02	(0.002)
**Pear**	0.06	(0.002)	1.12	(0.03)	6.54	(0.17)	2.74	(0.07)	0.17	(0.005)	N.D		1.23	(0.03)	1.40	(0.02)	0.03	(0.002)
**Tomato**	0.01	(0.001)	0.84	(0.001)	1.79	(0.001)	2.14	(0.002)	0.08	(0.001)	N.D		0.15	(0.000)	1.15	(0.03)	0.03	(0.005)
**White strawberry**	TR		0.71	(0.004)	2.96	(0.01)	2.45	(0.01)	0.19	(0.002)	TR		0.21	(0.004)	0.58	(0.007)	TR	

N.D: no detected; TR: traces.
