# Supplementary material for: Back to the Wastes: The Potential of Agri-Food Residues for Extracting Valuable Plant Cell Wall Polysaccharides
Source: Int J Mol Sci. 2025 May 21;26(10):4942. doi: 10.3390/ijms26104942 (PMC12112314; doi:10.3390/ijms26104942)
Supplement: Supplementary file 1 [file ijms-26-04942-s001.zip › ijms-3611596 Table S3.pdf]

Table S3. List of the antibodies used for this study.

Antibody	*Specificity*	*Isotype*	*Category*	*Host*	*Reference*
Pectins					
INRA-RU1	Anti-unbranched RG-I (requires at least 6 disaccharide backbone repeats for binding, maximal binding occurs to 7 disaccharide repeats)	IgM	Monoclonal	Mouse	[40]
CCRC-M36	Anti-unbranched RG-I (requires at least 3 disaccharide backbone repeats for binding)	IgM	Monoclonal	Mouse	[41]
LM6	Anti-arabinan (recognizes 4 subunits of 1,5-alpha-L-arabinan)	IgG	Monoclonal	Rat	[42]
JIM5	Homoglacturonan (partially methylesterified; **Me**(alpha)GalA1->4(alpha)GalA1->4(alpha)GalA1->4(alpha)GalA1->4(alpha)GalA1->4(alpha)**Me**GalA)	IgG	Monoclonal	Rat	[43,44]
JIM7	Homogalacturonan (highly methylesterified; GalA1->4GalA1->4**Me**GalA1->4**Me**GalA1->4**Me**GalA1->4GalA and **Me**GalA1->4GalA1->4**Me**GalA1->4GalA1->4**Me**GalA1->4GalA)	IgA	Monoclonal	Rat	[43,44]
2F4	Egg-box structure (dimeric association of pectic chains through calcium ions)	IgG	Monoclonal	Mouse	[45]
Hemicelluloses					
CCRC-M139	Anti-xylan (heteroxylan)	IgG	Monoclonal	Mouse	[41]
LM10	Anti-Heteroxylan; Beta-1,4-xylan	IgM	Monoclonal	Rat	[46]
LM15	Anti-xyloglucan (XXXG)	IgG	Monoclonal	Rat	[47]
LM24	Anti-xyloglucan (XLLG)	IgG	Monoclonal	Rat	[48]
LM25	Anti-xyloglucan (XXXG, XLLG, XXLG)	IgM	Monoclonal	Rat	[48]
